# The Solubility of False Diphtheritic Membranes

**Published:** 1868-10

**Authors:** 


THE SOLUBILITY OF FALSE DIPHTHERITIC MEMBRANES.—The *Journal de Chimie et de Pharmacie* for May contains a short review of the work of MM. Bricheteau and Adrian on this subject. One of the experiments is of interest: "A false tracheal membrane, weighing about twenty centigrammes, thick, resistant, and representing a square centimetre of surface, was placed in a tub containing about five grammes of water. To this was added two drops of lactic acid; the solution was then agitated. In two minutes the membrane began to disintegrate, and gave signs of dissolving. A few drops more of the acid brought about the complete solution of the membrane. A more complete result was obtained by using lime-water, so as to form lactate of lime. Solutions of potash and soda acted much less powerfully. Bromine water, chlorate of potassa, and common salt were all found less active in promoting solution of the membrane." The authors, therefore, recommend the solution of lactic acid as the best topical application to the false membranes of diphtheria.—*The Practitioner*, July, 1868—*Amer. Med. Journal.*

